# Measurements of Older Adults’ Physical Competence under the Concept of Physical Literacy: A Scoping Review

**DOI:** 10.3390/ijerph17186570

**Published:** 2020-09-09

**Authors:** Yan Huang, Kim-Wai Raymond Sum, Yi-Jian Yang, Nelson Chun-Yiu Yeung

**Affiliations:** 1 Department of Sports Science and Physical Education, The Chinese University of Hong Kong, Hong Kong, China; Hayleyhy@link.cuhk.edu.hk (Y.H.); yyang@cuhk.edu.hk (Y.-J.Y.); 2 The Jockey Club School of Public Health and Primary Care, The Chinese University of Hong Kong, Hong Kong, China; nelsonyeung@cuhk.edu.hk

**Keywords:** physical literacy, physical competence, measurements, older adults, scoping review

## Abstract

Physical literacy, especially in the fields of physical education and public health, has been gaining global interest in recent years. Applying an appropriate method to measure physical competence under the concept of physical literacy for older adults aligns with the goal of healthy aging. In this scoping review, we reflected on previous empirical studies regarding the measurements of physical competence among older adults holistically and systematically to identify and analyze gaps in the topic of “physical literacy” among older adults as a precursor to a systematic review. We searched five databases using the Preferred Reporting Items for Systematic Reviews and Meta-Analyses (PRISMA) for Protocols guidelines: (1) SPORTDiscus; (2) PubMed; (3) Scopus; (4) ScienceDirect; and (5) Web of Science. There were 29 studies included in our thematic analysis. Through our review, we found that 73% of the mean age of the participants comprised older baby boomers who were from 65–74 years old as aging continues. Therefore, more effort should be made in developing physical literacy for older adults with the goal of health promotion. Our results showed that most studies adopted both self-reported and objective measures, in which objective measures were widely embraced by scholars in the measurement, while self-reported measures were encouraged to be included in the assessment as well. Using assessment tools to measure a combination of actual physical competence and perceived physical competence is recommended in the measurement of physical competence, especially in older adults. In addition, other elements of physical literacy should be taken into account when measuring physical competency in older adults. For future implementation, when framing the model to chart physical literacy for older adults, it is important to review the definition again and adopt a holistic measurement system including every aspect of physical literacy.

## 1. Introduction

### 1.1. Physical Literacy

Terms that have been used to describe embodied dimensions of physical literacy include physical activity, physically able, physically educated, etc., of which physical activity is the most recognized term used by scholars worldwide [1]. However, these terms are prone to a misunderstanding of physical and mental dualism, and the neglect of the embodied capacity of each individual by viewing the body as an object in sports, schools, or manual work settings [2]. In recent years, a holistic concept of physical literacy has been a focus in international literature. Although the first use of this term in academia could date back to 1938 in the *Journal of Health and Physical Education* [3], physical literacy was not embraced worldwide until there was a debate about physical literacy based on monism [4], when the terminology of physical literacy received more attention from researchers, policymakers, and stakeholders worldwide. Influenced by different natural and cultural backgrounds, the interpretation of the definition of physical literacy differs. In the US, physical literacy is interpreted as the ability to move with competence and confidence [5]. In Australia, the understanding of physical literacy covers physical, psychological, cognitive and social domains [6]. However, in a previous systematic review of definitions of physical literacy, 70% of the papers endorsed the concept raised by Whitehead [7], which was also adopted by The International Physical Literacy Association. As applied to each individual, a widely adopted physical literacy can be described as the motivation, confidence, physical competence, knowledge and understanding to value and take responsibility for engaging in physical activities for life [8]. Physical literacy covers physical, cognitive, and affective domains, which has encouraged cooperation in the operationalization of physical literacy and the implementation of promotional programs, especially among the younger generation. 

Physical literacy could be described as a disposition or an attitude acquired by an individual throughout a lifetime, a lifelong journey beginning in early childhood until old age [9]. Physical literacy is relevant to everyone no matter what age and ability. A physical literacy journey refers to an individual’s ongoing commitment to participate in physical activity [10]. Therefore, the acquisition of physical literacy is continuous throughout an individual’s life [2]. Physically literate older adults are able to make physical adaptations especially when facing the challenges that come with injury, chronic disease, and aging, which allows them to remain independent for a longer time than those who are less active [2]. Successful agers refer to those who are self-supportive with varying needs and capacities, and who can make changes to their environments and maintain the functional ability to do the things they value [11]. From a physical literacy perspective, successful agers “compensate and modify their activity (age adaptation and physical competence)” by “optimizing choices (motivation and movement enjoyment)”, thereby “maximizing success (confidence) and maintaining higher levels of functioning” across all dimensions [12]. Developing and maintaining physical literacy is consistent with the goal of healthy aging, and optimizes opportunities for good health at all stages of life. Therefore, it is important to highlight that an active start can contribute to sustained independence, and improved population health and well-being [13]. Developing physical literacy for older adults needs to be emphasized.

### 1.2. Physical Competence 

Physical competence, as one of the elements within physical literacy, could be described as the proficiency in movement, capacities and developed movement patterns afforded by an individual’s ability [14] (p. 204). This concept was enriched by emphasizing physical competence as movement patterns that constitute the foundation of all movement within a wide range of environments [15] (p. 78). To be more accurate, physical competence could be further categorized as simple capacities (e.g., core stability); combined capacities (e.g., poise which incorporates both balance and core stability) and complex capacities (e.g., bilateral coordination) [2]. 

### 1.3. Measurements of Older Adults’ Physical Competence under the Concept of Physical Literacy 

The nature of and theoretical basis for physical literacy, together with its consistent practice even through changed circumstances and needs, are decisive in its critical role in a person’s life. With the evidence indicating a positive relationship between physical literacy and healthy aging, the development of physical literacy for older adults should be introduced and included as part of public health, specifically in areas facing a rapidly aging population. To date, however, there has not been a systematic approach to conceptualizing physical literacy for older adults [16].

Measurements could make the concept tangible to multiple stakeholders and allow researchers to understand what strategies are most effective in the process of developing physical literacy and health promotion [17]. There are already physical literacy assessment tools designed for and applied to children and adolescents in different areas around the world. The Canadian Assessment of Physical Literacy-2 (CAPL-2) [18] was designed to measure the actual physical literacy of children in Canada, and this has also been translated into Chinese, and embraced by researchers in Hong Kong [19].

However, given that adolescents and older adults are at the two ends of the physical literacy journey with different characteristics of physical function, the objectives and requirements for the measurements of physical literacy for these two groups of people vary. Accordingly, the measurement of physical literacy should be differentiated and more targeted to different populations. Measurement means the process of quantifying objectives or recording the observations in qualitative research [20]. According to [21], the measurement of physical literacy mainly depends on how we define it. The definition of physical literacy that we are using is the Whiteheadian concept [8] (p. 8), which includes physical, cognitive, and affective domains, with four core elements (i.e., physical competence, motivation, confidence, knowledge, and understanding). Therefore, an optimal measurement design for older adults should be a holistic approach including all the core elements.

Physical competence is also the core value of physical literacy, and a fundamental aspect of being human [22]. Therefore, the measurement of physical competence was the topic of this scoping review. In previous research, the assessment of physical competence for older adults was conducted to explore the relationships between perceived physical competence and physical activity engagement [23] or to predict the risk of injuries or diseases under the section of rehabilitation and public health [24]. Although these studies have not indicated that the physical competence assessed was under the concept of physical literacy, due to the aforementioned relationship between physical literacy and healthy aging, it is evident that the core abilities of physical competence within physical literacy are in line with the ones assessed by researchers conducted out of health promotion.

Thus, it is essential to identify a proper approach to measure physical competence under the concept of physical literacy for older adults through a scoping review. In this way, we hope to contribute to the development of physical literacy and health promotion.

### 1.4. Objectives

Scoping reviews are embraced for their nature of examining the range and extent of research in emerging fields [25]. We conducted this scoping review to identify and analyze gaps in the topic of “physical literacy” among older adults as a precursor to a systematic review. In this scoping review, we explored the measurements of older adults’ physical competence under the concept of physical literacy, with the following specific aims:

1. To reflect on previous empirical studies regarding the measurements of physical competence under the concept of physical literacy for older adults holistically and systematically;
2. To critically characterize and evaluate previous measurements practice;
3. To propose an appropriate instrument to evaluate physical competence for older adults that can contribute to further implementation for health promotion.

## 2. Methods

This scoping review followed the Preferred Reporting Items for Systematic Reviews and Meta-Analyses (PRISMA) extension for scoping reviews statement [26]. PRISMA guidelines have been increasingly important especially in health-related fields. These have been helpful in previous systematic reviews on physical literacy as well [1,7]. Thematic synthesis was adopted when exploring and organizing the empirical findings. Supplementary Table S1 detailed the example of the search queries for the database. And Supplementary Table S2 displayed the PRISMA-ScR Checklist.

### 2.1. Eligibility Criteria

The inclusion criteria in the scoping review were: (1) peer-reviewed papers; (2) publication time was set from 1 January 2001–31 December 2019 to reflect contemporary approaches to delivery and practice, for 2001 was the year that physical literacy started to attract empirical attention. (3) publications in the English language. The following exclusion criteria were adopted: (1) papers without empirical findings; (2) conference reports, editor letters, and review studies; (3) papers not using a validated measurement tool; and (4) multiple publications on the same participants. Additional records were selected by identifying sources from the reference lists of the records identified through database searching.

### 2.2. Information Sources and Study Records

**Figure 1:**
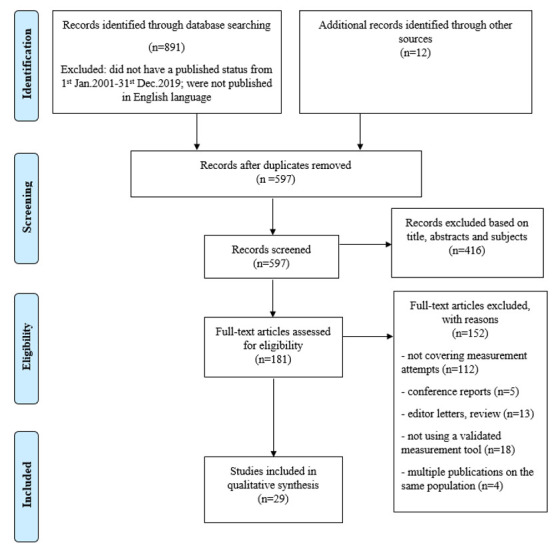
Preferred reporting items for systematic reviews and meta-analysis flow diagram.

Figure 1 shows the process of the selection of eligible papers. Five databases were searched using the PRISMA for Protocols guidelines: (1) SPORTDiscus; (2) PubMed; (3) Scopus; (4) ScienceDirect; and (5) Web of Science. These databases cover reports on sport and health, which increased the probability that related research was included. English-language, peer-reviewed published papers containing empirical studies of measurements of physical competence within physical literacy were analyzed using inductive thematic analysis. A Boolean logic combinations search strategy was adopted within the electronic databases, including “physical competence” with older adults, elderly, and old people. Inverted commas were applied to the term “physical competence” to ensure searches would find papers concerning physical competence as opposed to searches related to ‘physical’ and ‘competence’. Owing to the limited empirical research to assess physical competence under the framework of physical literacy, papers with related constructs and understanding were also included in this scoping review (e.g., physical capacity and physical capability). The time set was from the start of 2001 to the end of 2019, and English language, peer-reviewed, and journal filter boxes were marked on all searches to ensure only these papers would appear in the results.

There were 891 records identified through the database search and an additional 12 records were retrieved from the reference lists within the 891 articles above. After duplicates were removed, 597 articles remained in the screening process. Two authors conducted the initial study selection by assessing the titles, abstracts, and subjects independently, and when discrepancies occurred, the third author was involved in the discussion until a consensus was reached. A total of 416 records were excluded based on the title, abstracts, and subjects that did not meet our inclusion criteria. In the end, there were 181 full-text articles assessed for eligibility, with 29 published articles included in the final qualitative synthesis.

### 2.3. Data Synthesis

To have a better understanding and comparison of the measurements of physical competence within physical literacy for older adults, we conducted a qualitative synthesis using thematic analysis [1]. Thematic analysis was adopted to distinguish common categories among these 29 articles. To start with, we performed inductive thematic analysis to extract, label, and evaluate data from the 29 articles. Characteristics of studies including country of origin, environment, study design and sample size enrolled, participants’ characteristics including gender, mean age and health status, outcome measures, strengths, and limitations of measurements in relation to physical competence within physical literacy for older adults were extracted from these articles respectively. This process summarized the key features of included studies. After thorough and further interpretative coding [27], more specific trend of data was generated. Initial codes were obtained inductively under the heading of “Self-reported” and “Objective”. Through carefully reading the papers and highlighting the keywords concerning the two higher-order themes, sub-themes and core categories were identified. There were six sub-themes and 23 measurement tools identified through the analysis (Table 1).

## 3. Results

This scoping review seeks to reflect on previous empirical studies regarding the measurements of physical competence under the concept of physical literacy for older adults. Through searches from multiple databases and thematic synthesis, the results are presented as follows.

### 3.1. Summary of Studies

Table 1 summarizes the coding result of the thematic analysis. There were 28 articles using both self-reported and objective measures; 19 articles applied objective measures, and only one article solely used self-reported measures. For the self-reported higher order theme, seven core categories were presented under the sub-themes of perceived physical competence, health-related quality of life, and physical capabilities. The Physical Self-perception Profile [28,29] was introduced in two studies to evaluate perceived physical competence. Questionnaires are common tools to measure health-related quality of life. Five studies adopted the Medical Outcomes Study Short Form–36 Questionnaire [30,31,32,33,34]. There were also the Nottingham Health Profile Questionnaire [35] and the World Health Organization Quality of Life-OLD (WHO QOL-OLD) Questionnaire [36]. Under the sub-theme of physical capabilities, the assessment tools covered instrumental activities of daily living [36,37], basic activities of daily living [37] and life-space assessment [37].

For the objective higher-order theme, there were three sub-themes with 17 core categories: simple capacities, combined capacities, and complex capacities. Most researchers adopted the gait speed measures [24,30,35,38,39,40,41] to assess older adults’ simple capacities. The timed up and go test [31,42,43], sit-to-stand test [32,44], stabilometer and posture meter platforms [40,45] and the one-repetition maximum test [46,47] were also implemented in more than one study. While under the sub-theme of combined capacities, the single-leg stance [33,40,42,48] was embraced by most researchers among all the five assessment tools. The Senior Fitness Test [49,50,51,52] and the Short Physical Performance Battery [34,37,53] were the most common measures to assess complex capacities of older adults.

### 3.2. Characteristics of Studies and Their Participants

As illustrated in Table 2, seven of these studies were conducted in Brazil, five in the U.S. and three in China. For the study settings, nine of the studies were community-based research and six were hospital-based. Three were care center-based studies. Sixteen papers were observational studies while randomized controlled trials (RCTs) were adopted in 13 studies. With regards to the sample size enrolled, there were 17 studies with under 100 participants, five had over 300 participants and seven studies had between 100–300 participants.

Among all the 29 studies identified, there were a total of 759 patients. Over half of the participants were physically active or in a good state of health that allowed them to finish their study without any difficulties. In terms of gender, most of the studies reported more female participants. Regarding age, 73% of the mean age of the participants comprised older baby boomers who were from 65–74 years old (Table 3).

## 4. Discussion

To date, there is limited empirical research to assess physical competence under the framework of physical literacy. Therefore, papers with related constructs and understanding were also included in this scoping review (e.g., physical capacity and physical capability). According to the coding themes of thematic analysis, critical analysis of these two higher-order coding themes was conducted, respectively (Table 4 and Table 5). Table 4 shows the critical analysis of self-reported measures of physical competence within physical literacy for older adults while Table 5 focuses on objective measures. The following section will mainly discuss the strengths and limitations of each outcome measure included.

### 4.1. Self-Reported Measures

Self-reported measures are often adopted together with objective measures. In articles included in the scoping review, the questionnaire was the main instrument applied in the self-reported measures (Table 4). Among all the self-reported measures, the Medical Outcomes Study Short Form–36 Questionnaire [30,31,32,33,34] was introduced in the assessment of physical competence within physical literacy. It is a widely used health-related quality of life index with high validity. While its wide coverage on the health-related issues is of significance, the time consumed on the completion of the 36-item questionnaire may reduce reliability, especially for older adults. The Nottingham Health Profile Questionnaire [35] shared the same strengths and limitations in its comprehensive sub-domains measures and large-scale of 38 measurement items, containing six subdomains: energy, pain, emotional reactions, sleep, social insulation, and physical activity.

Basic activities of daily living [37], instrumental activities of daily living [36,37] and life-space assessment [37] took daily activities (e.g., using the toilet, dressing, transferring and bathing) into account when measuring the physical competence of older adults, which is actually an emphasis on cognitive functions. It is notable that the life-space assessment [37] documented mobility within participants’ homes and communities, which showed the influence of the living environment in the process. All these measures may be conducted in the evaluation of the environment prior to the implementation.

The World Health Organization Quality of Life-OLD (WHO QOL-OLD) Questionnaire [36] and Physical Self-perception Profile [28,29] emphasized the psychometric domain, however, the Physical Self-perception Profile was designed specifically to assess perceived physical competence, which covers four sub-scales (physical confidence, body, strength and physical self-worth), and participants’ awareness of self-functional dimension could be highlighted easily when they were involved in this process.

### 4.2. Objective Measures

In line with the categories raised by Murdoch and Whitehead in 2010, the outcome measures could be further divided into simple capacities, combined capacities, and complex capacities.

In these studies, simple capacities such as mobility, strength, aerobic fitness, flexibility, and balance were assessed through simple but effective instruments. The gait speed measures [24,30,35,38,39,40,41] were embraced by most researchers for their function serving as a core indicator of health and function in ageing and disease. But this was often simply introduced in clinical settings to monitor and evaluate changes. The timed up and go test [31,42,43] was a core predictor of the risk of fall. However, the assessors only looked at a recorded time, so the results may not indicate a patient’s movements and whether the chair was used in the test, perhaps leading to unpredictable results as well. The sit-to-stand test [32,44] and the one-repetition maximum test [40,45] were both easy to operate to assess the strength of the older adults. The former one was limited to high-functioning elderly in the clinical setting, while the latter one risked participant injury due to the extent of warm-up and the time consumed. The handgrip strength test [33], functional upper body strength test [43] and counter movement jump [42] were reliable when measuring upper or lower body strength. However, the result may be affected by the individual variation and the surface of the ground.

When the exercise stress test [31] and stabilometer and posture meter platforms [40,45] are conducted, the special settings and environment should be emphasized. There is a need for a treadmill or exercise bike when the former test is conducted. Also, the risk of getting injured while completing the trunk flexion test [54] needs to be stressed as well.

As for the combined capacities, the single-leg stance [33,40,42,48] and timed tests of standing balance [38] focus on the assessment of balance and strength of older adults, which are easy to monitor and have many variations for specialized needs.

However, the surface of the ground and aid equipment may affect the accuracy of the results. Balance together with mobility also could be evaluated through the Berg Balance Scale [35], a standard 14-item list with each item consisting of a five-point ordinal scale, and the Performance-oriented Mobility Assessment [36,55], a task-oriented test. They were often introduced in clinical settings. Stair climbing [32,43] was implemented among the older adults for its efficacy and economic considerations. There is, however, a risk of knee pain arising from this process.

There were two comprehensive and well-conducted measures available and reliable for assessing physical competence within physical literacy for the older population. The Senior Fitness Test [49,50,51,52] and the Short Physical Performance Battery [34,37,53] were reported to be able to measure the complex capabilities with excellent test–retest reliability especially in community-dwelling older adults. These two kits of assessment tools examine the ability of strength, mobility, balance, flexibility, and aerobic fitness for the older adults. Some tasks may overlap; however, considering that they measure the basic but comprehensive physical movement skills that an older adult needs to maintain an independent life, they were common objective measurement adopted by researchers worldwide.

### 4.3. Limitations

While the objective of this scoping review is to reflect upon and critique previous findings and to find a proper method to assess physical competence within physical literacy looking at different types of studies, this study did not conduct a meta-analysis of the efficacy of the interventions included. Papers written in English were the sole sources, and that could be a limitation for this study as it may not be generalizable to the practice in other language settings. Owing to the limited empirical research on the element of physical competence within physical literacy, we also included the related concepts (e.g., physical capacity and physical capability).

To our knowledge, this study was the first scoping review in the field of measurement of physical competence under the concept of physical literacy among the older population, in which the effort highlights the gaps in the development of physical literacy. Our review is expected to offer us an opportunity to understand the practice of the physical literacy journey and the emphasis on health promotion.

### 4.4. Conclusions

Some developed areas are now impacted by the burden of an aging population. The number of older baby boomers (born between 1946–1955) is rising rapidly as aging continues. It is expected that this population will continue to age, and its rate will accelerate significantly in the next 20 years, particularly in the coming 10 years. By 2030, the percentage of people aged 60 years or over is projected to account for 33.6% of the total population in Hong Kong [56]. This continuing situation may cause heavy burdens on public services and potential problems such as a shortage of medical resources. However, there has been limited attention to the positioning of physical literacy in the field of public health [19]. More effort should be made in developing physical literacy for older adults with the goal of health promotion.

After we reflected on previous empirical studies regarding the measurements of physical competence under the concept of physical literacy for older adults, we found that objective measures were widely adopted by scholars in the measurement of physical competence or related concepts within physical literacy for older adults. Self-reported measures could be included in the assessment as well, for elderly participants actively pursuing health and functional goals rather than personal feelings about sports skills [28].

In the studies included, none measured physical competence under the concept of physical literacy. For future implementation, it is of great significance that applying an appropriate method to measure physical competence within physical literacy for older adults not only lies in the nature of the physical literacy journey and its contribution to the goal of healthy aging, but could also be an indicator of the health outcomes among older people [36,55]. For the older population, adequate balance, strength, and mobility play a crucial role in retaining and maintaining an independent lifestyle. All components identified within the concept of physical literacy, which we suggest includes a combined and comprehensive kit of assessment of actual physical competence (e.g., Senior Fitness Test) and perceived physical competence (e.g., Physical Self-perception Profile) could be implemented in the measurement of physical competence within physical literacy especially for the older baby boomers. And when we evaluate the measurement level, it should be noted that the goal is to make progress, not to master every aspect of physical competence.

However, physical competence can never be the sole constituent of physical literacy [57]. Physical, cognitive, and affective domains within physical literacy should be taken into account in future evaluations. Recommendations are warranted to include selected, specific exercises that can support the construct of physical literacy for older adults [12]. Therefore, when framing the model to chart physical literacy for the elderly, it is important to review the definition again and adopt a holistic measurement system including every aspect of physical literacy.

## Figures and Tables

**Table 1:** Thematic analysis of the measures of physical competence within physical literacy for older adults.

Higher-Order Themes	Sub-Themes	Measures
**Self-Reported**	Perceived Physical Competence (2) ^a^	Physical Self-perception Profile (2)
	Health-related Quality of Life (7)	Medical Outcomes Study Short Form—36 Questionnaire (5)
		Nottingham Health Profile Questionnaire (1)
		World Health Organization Quality of Life-OLD (WHOQOL-OLD) Questionnaire (1)
	Physical Capabilities (3)	Instrumental Activities of Daily Living (2)
		Basic Activities of Daily Living (1)
		Life-Space Assessment (1)
**Objective**	Simple Capacities (18)	Gait Speed Measures (7)
		Timed Up and Go Test (3)
		Sit-to-stand Test (2)
		Stabilometer and Posture Meter Platforms (2)
		The One-repetition Maximum Test (2)
		Exercise Stress Test (1)
		Handgrip Strength Test (1)
		Functional Upper Body Strength Test (1)
		Counter Movement Jump (1)
		Trunk Flexion Test (1)
	Combined Capacities (10)	Single-leg Stance (4)
		Performance-oriented Mobility Assessment (2)
		Stair Climbing (2)
		Berg Balance Scale (1)
		Timed Tests of Standing Balance (1)
	Complex Capacities (7)	Senior Fitness Test (4)
		The Short Physical Performance Battery (3)

^a^ Numbers in parenthesis represent the number of papers that referred to the core categories apparent out of the 29 papers.

**Table 2:** Study characteristics.

Characteristics	Value
**Country of origin**	
Brazil	(7) ^a^
United States	−5
China	−3
Norway	−2
Japan	−2
Turkey	−2
Others	−8
**Environment**	
Community-based	−9
Hospital-based	−6
Care center-based	−3
Others	−11
**Study design**	
Observational studies	−16
Randomized controlled trials (RCTs)	−13
**Sample size enrolled**	
1–100	−17
101–200	−4
201–300	−3
300 above	−5

^a^ Numbers in parenthesis represent the number of papers that referred to the corresponding characteristic apparent out of the 29 papers.

**Table 3:** Participants characteristics.

Characteristics	Value
**Female**	3235 (62) ^a^
**Mean age**	
Under 65	421 (8)
65–74	(73)
75 above	(19)
**Health status**	
Physically active	2798 (53)
Patients	759
Others	1701

^a^ Data presented as n (%) of study participants.

**Table 4:** Critical analysis of self-reported measures of physical competence within physical literacy for older adults.

Measures (No. of Papers)	Design (No. of Papers)	Outcome Measures Assessed (Physical Domain)	Strengths	Limitations
Physical Self-perception Profile (2) [28,29]	Observational studies (2)	Perceived Physical Competence	Covers physical confidence, body, strength and physical self-worth; Awareness of self-functional dimension highlighted easily.	Too much emphasis on the psychometric domain.
Medical Outcomes Study Short Form—36 Questionnaire (5) [30,31,32,33,34]	Observational study (1); RCTs (4)	Physical Function	A widely used health-related quality of life index.	The 36-item questionnaire takes a long time to complete, which may reduce the reliability especially for older adults.
Nottingham Health Profile Questionnaire (1) [35]	RCT (1)	Physical Capabilities	Contains comprehensive sub-dimensions: Energy, pain, emotional reactions, sleep, social insulation and physical activity.	The 38-item questionnaire takes a long time to complete, which may reduce the reliability especially for older adults.
WHOQOL-OLD Questionnaire (1) [36]	Observational study (1)	Physical Capacity	Modified specially for elderly,6 facets, 24 items with high reliability.	Lack of an explicit connection with the physical domain.
Basic Activities of Daily Living (1) [36]	Observational study (1)	Physical Function	Contains 5 items: eating, using the toilet, dressing, transferring and bathing, daily physical activities considered.	Lack of specific physical training tasks.
Instrumental Activities of Daily Living (2) [36,37]	Observational studies (2)	Physical Function	Contains daily physical activities: using the telephone, managing money, preparing meals, doing light housework, etc.	Too much emphasis on the cognitive domain.
Life-Space Assessment (1) [37]	Observational study (1)	Physical Mobility	Document participants’ mobility within their home and community.	Omits other parts of physical function.

**Table 5:** Critical analysis of the objective measures of physical competence within physical literacy for older adults.

Measures (No. of Papers)	Design (No. of Papers)	Main Outcome Measures Assessed		Strengths	Limitations
Gait Speed Measures (7) [24,30,35,38,39,40,41]	Observational studies (6); RCT (1)	Simple Capacities (18)	Mobility	Serving as a core indicator of health and function in ageing and disease.	Short timed tests in clinical practice, for both screening purposes and to evaluate change.
Timed Up and Go Test (3) [31,42,43]	Observational study (1); RCTs (2)		Mobility	Serving as a core predictor of the risk of fall.	Omits patient’s movements; The chair used may impact the results.
Sit-to-stand Test (2) [32,44]	Observational study (1); RCT (1)		Strength	Easy to operate and monitor.	Restricted to high-functioning elderly in clinical setting.
The One-repetition Maximum Test (2) [46,47]	RCTs (2)		Strength	Effective in measuring the Maximum strength.	Warm-up needed, time consuming, high risk in getting injured.
Handgrip Strength Test (1) [33]	RCT (1)		Strength	Simple and commonly used test of general strength level.	A dynamometer needed and will affect accuracy.
Functional Upper Body Strength Test (1) [43]	RCT (1)		Strength	Comprehensive test on upper body strength.	There may be individual variation in reporting.
Counter Movement Jump (1) [42]	Observational study (1)		Strength	No equipment needed, measuring lower body strength.	Surface may affect the assessment.
Exercise Stress Test (1) [31]	RCT (1)		Aerobic Fitness	A treadmill or exercise bike needed, safe setting, accurate data.	Limited equipment.
Trunk Flexion Test (1) [54]	Observational study (1)		Flexibility	Focusing on low back and hamstring flexibility.	Risk of getting injured during the process.
Stabilometer and Posture Meter Platforms (2) [40,45]	Observational study (1); RCT (1)		Balance	Measuring balance with low risk in getting injured.	Limited special extent.
Single-leg Stance (4) [33,40,42,48]	Observational studies (3); RCT (1)	Combined Capacities (10)	Balance & Strength	Simple and can be conducted using many variations.	Surface and Aid equipment may affect accuracy.
Timed Tests of Standing Balance (1) [38]	RCT (1)		Balance & Strength	Easy to administer and cost effective.	Surface and Aid equipment may affect accuracy.
Berg Balance Scale (1) [35]	Observational study (1)		Balance & Mobility	A standard 14-item list with each item consisting of a five-point ordinal scale.	Not appropriate to those who are ataxic. Time consuming.
Performance-oriented Mobility Assessment (2) [36,55]	Observational studies (2)		Balance & Mobility	Task-oriented test, easily administered, good indicator of the fall risk.	Often adopted in clinical setting.
Stair Climbing (2) [32,43]	RCTs (2)		Balance, Strength & Agility	Simple to administer, economic.	May increase risk of knee pain
Senior Fitness Test (4) [49,50,51,52]	Observational studies (2); RCTs (2)	Complex Capacities (7)	Aerobic Fitness, Strength, Flexibility & Balance	Using minimal and inexpensive equipment	A combination of simple capacity tests omits the coordination.
The Short Physical Performance Battery (3) [34,37,53]	Observational study (1); RCTs (2)		Mobility, Strength & Balance	Excellent test-retest reliability in community-dwelling older adults. Aids in monitoring function of elderly.	A combination of simple capacity tests, some overlaps.

## Supplementary Materials

The following are available online at https://www.mdpi.com/1660-4601/17/18/6570/s1: Supplementary Table S1: Full electronic search strategy for SPORTDiscus; Supplementary Table S2: Preferred Reporting Items for Systematic reviews and Meta-Analyses extension for Scoping Reviews (PRISMA-ScR) Checklist.
