# Supplementary material for: Measurements of Older Adults’ Physical Competence under the Concept of Physical Literacy: A Scoping Review
Source: Int J Environ Res Public Health. 2020 Sep 9;17(18):6570. doi: 10.3390/ijerph17186570 (PMC7558186; doi:10.3390/ijerph17186570)
Supplement: Supplementary file 1 [file ijerph-17-06570-s001.zip › Supplementary - Table S1.pdf]

Full electronic search strategy for SPORTDiscus

1. Log into database (SPORTDiscus), and insert keywords into search field (i.e., “physical competence” OR “physical capacity” AND “older adults” OR “older people” OR “elderly”). Ensure inverted commas are inserted to the keywords.

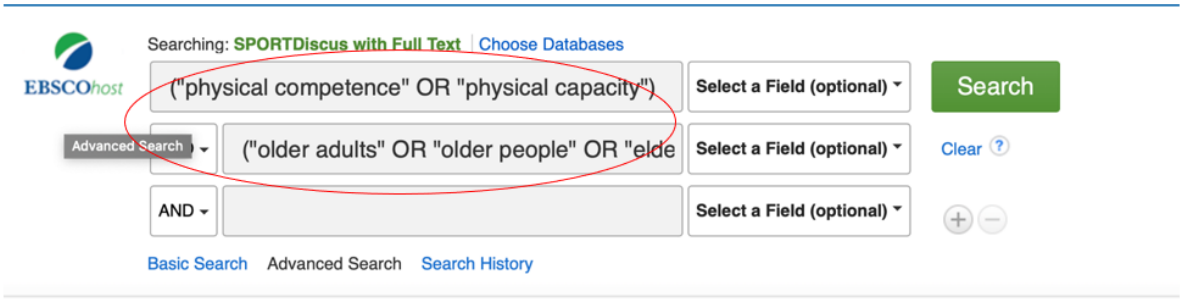

2. Select the ‘Peer reviewed’ and ‘English language’ limits. And time was set from January 2001 to December 2019. Press search to view results.

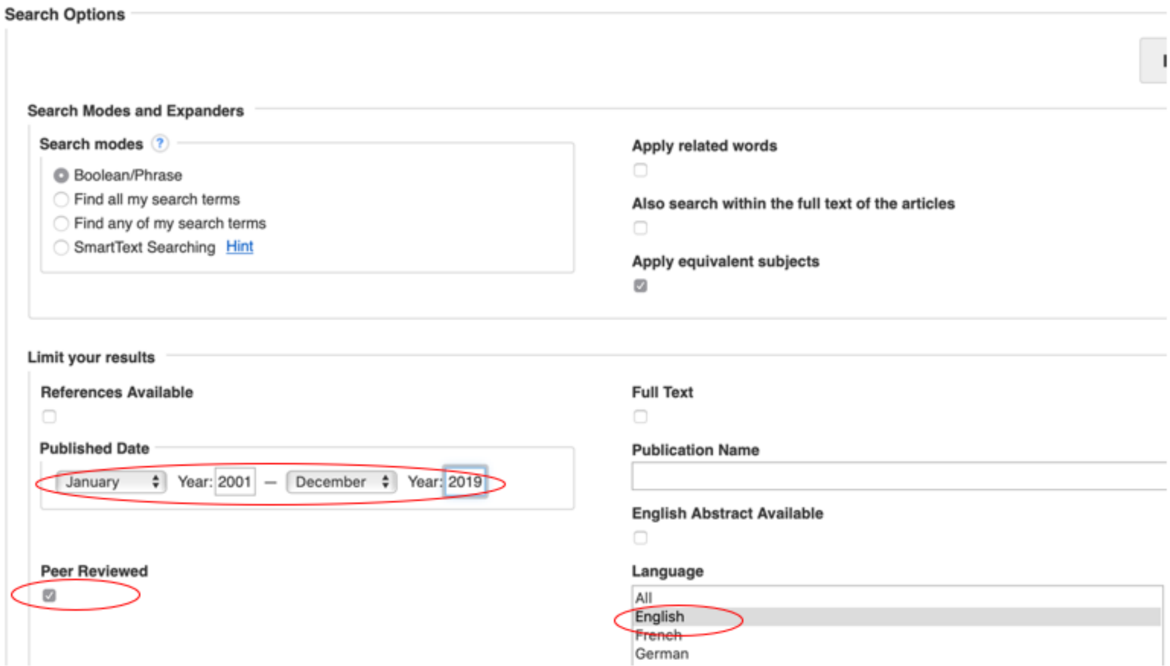

3. The number of results will appear (i.e., 56 results).

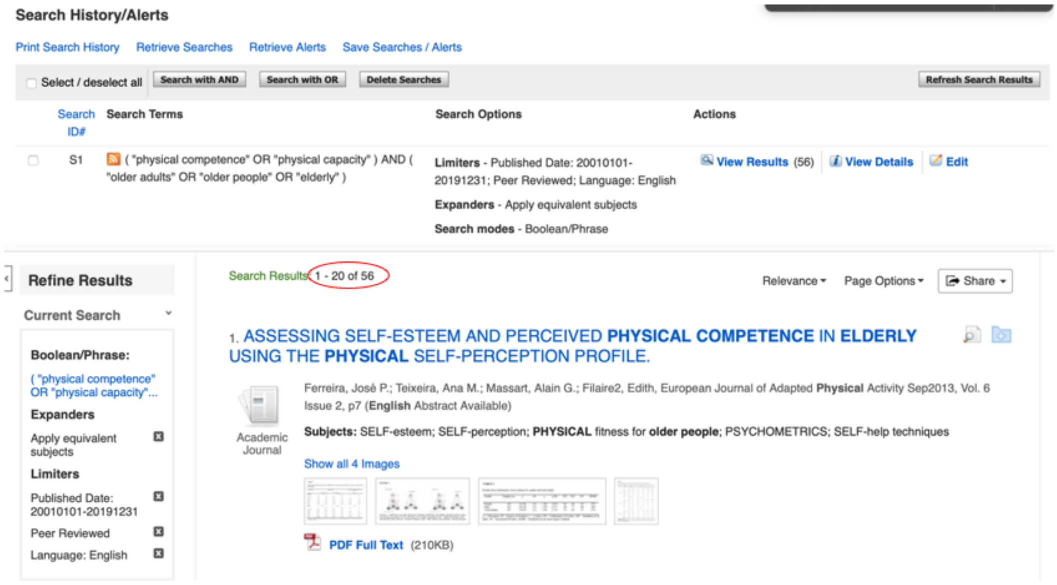

4. Copy and paste each result (reference) into Mendeley, and after duplicates removed, there were 53 results left in total.

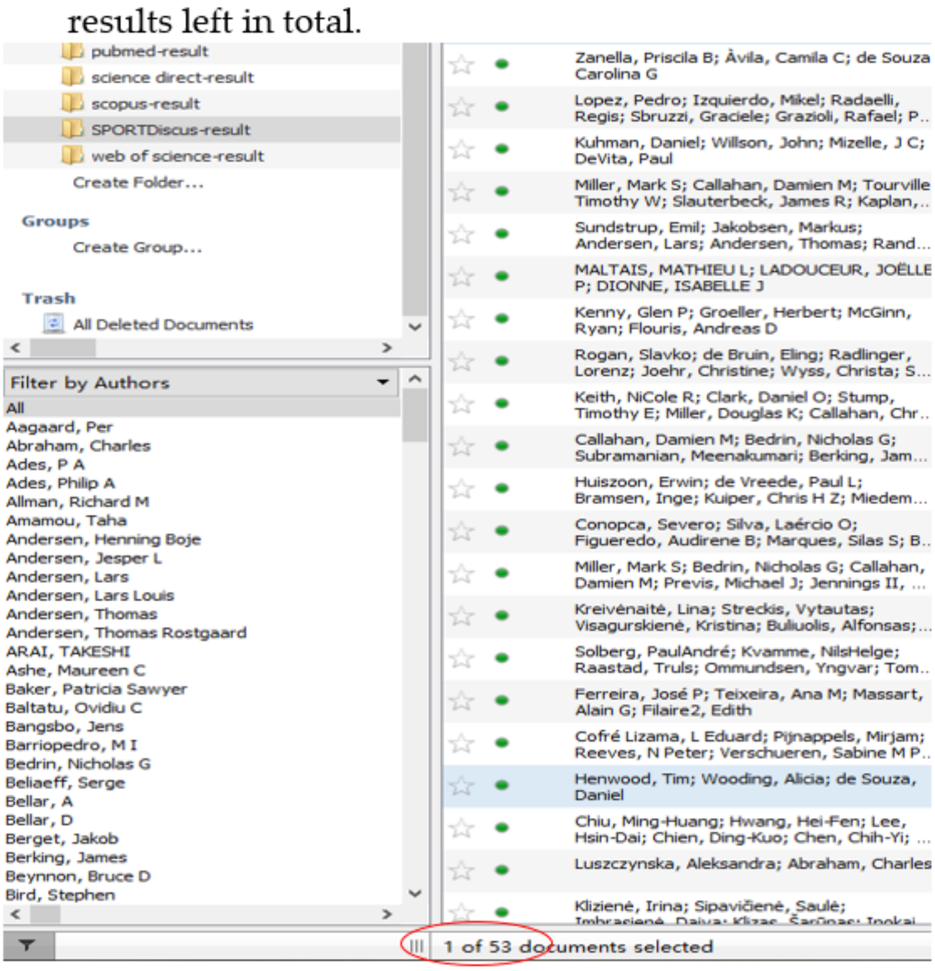

5. Repeat process consistently with other databases (PubMed; Scopus; ScienceDirect; Web of Science).
